# Decoupling Analysis of Rural Population Change and Rural Electricity Consumption Change in China

**DOI:** 10.3390/ijerph19116676

**Published:** 2022-05-30

**Authors:** Xuechao Xia, Hui Sun, Zedong Yang, Weipeng Yuan, Dianyuan Ma

**Affiliations:** 1 Center for Innovation Management Research of Xinjiang, No.666 Shengli Road, Urumqi 830046, China; xiaxuechao99@163.com (X.X.); 15066198893@163.com (Z.Y.); ywpywp@163.com (W.Y.); madianyuan2022@163.com (D.M.); 2 School of Economics and Management, Xinjiang University, No.666 Shengli Road, Urumqi 830046, China

**Keywords:** rural permanent population, electricity consumption, decoupling, coordination

## Abstract

With the accelerated development of urbanization in China, rural permanent population has declined, while rural electricity consumption has increased, resulting in a significant waste of electricity resources. Based on the provincial panel data of China from 2007 to 2020, this paper comprehensively used the decoupling model and the coordination degree model to analyze the temporal change characteristics, spatial distribution characteristics, and the degree of deviation of rural permanent population and rural electricity consumption. Firstly, according to the decoupling model, the type of decoupling between rural electricity consumption and rural permanent population was strong negative decoupling. At the provincial level, Beijing and Tibet belonged to expanding negative decoupling. Tianjin and Liaoning belonged to recession link. The other 27 provinces, including Hebei, Shanxi, and Shandong, belonged to strong negative decoupling. Secondly, according to the coordination degree model, the coordination type of the national rural permanent population and rural electricity consumption was uncoordinated. The areas that can be coordinated include 20 provinces, including Shanghai, Inner Mongolia, Jilin, Jiangsu, Anhui, Fujian, and Jiangxi. The basic coordination areas included Beijing and Tibet. Finally, according to the comprehensive measurement model, the provinces with strong negative decoupling included Shanxi, Zhejiang, and Chongqing. Sichuan, Hebei, Shandong, and Shaanxi belonged to moderately strong negative decoupling groups.

## 1. Introduction

Since China’s reform and opening up in 1978, China has accelerated the process of urbanization, and rural population has been pouring into cities. According to the seventh national census data of 11 May 2021, the urbanization rate in China was 63.89% by 2020. Urbanization has promoted social development in China, but it also led to a rapid decline in the rural population [1]. According to China Statistical Yearbook 2007 and China Statistical Yearbook 2020, the permanent population in China’s rural areas decreased from 714.96 million in 2007 to 509.92 million in 2020, a decrease of 200.54 million, accounting for 28% of the number of rural permanent population.

Population mobility and agglomeration are often accompanied by changes in energy consumption patterns [2,3]. Electricity, as an essential energy closely related to people’s lives, cannot be ignored in the process of urbanization in China [4]. According to China Energy Statistical Yearbook 2007 and China Energy Statistical Yearbook 2020, China’s rural electricity consumption has increased from 550.99 million kWh in 2007 to 971.72 million kWh in 2020. This shows that China’s rural areas have not reduced electricity consumption due to the decline of the permanent population, and even the phenomenon of “increasing electricity after people leave” has resulted in a serious waste of electricity resources. Generally speaking, electricity consumption increased with the increase of population [5,6,7], but there was a serious imbalance between the permanent population and electricity consumption in China’s rural areas. In addition, due to the wide variation of rural areas in China, changes in rural permanent population and rural electricity consumption varied from province to province. Therefore, what were the specific trends in the permanent population and electricity consumption in China’s rural areas? What were the types of decoupling between rural permanent population and electricity consumption in different regions? What were the background causes of changes in rural resident population and changes in electricity consumption in each region? In the context of global energy shortages and inequitable distribution of energy [8,9], it is essential to explore the decoupling relationship between rural permanent population and electricity consumption in various provinces, which not only reveal the relationship between the two, but also help to provide policy recommendations for the government to formulate reasonable power resource policies. Therefore, this paper used geographic information system (GIS) technology, combined with quantitative analysis decoupling model and coordination degree model, to analyze the degree of decoupling between China’s rural permanent population and rural electricity consumption. Next, we verified and supplemented the research content of population and power energy, which enriched the research methods of the relationship between rural permanent population and rural electricity. Specifically, the research contributions of this paper are that we: (1) revealed the temporal variation characteristics and spatial distribution characteristics of China’s rural permanent population and rural electricity consumption, (2) evaluated and analyzed the decoupling relationship between China’s rural population and rural electricity consumption, and (3) analyzed the coordination relationship between rural population and rural electricity consumption according to the coordination degree model. The rest of this paper is structured as follows. The second part is the current research status of China’s rural population change and electricity consumption change, and comprehensively analyzes the progress and shortcomings of existing research. The third part introduces the data sources and the decoupling and coordination models used in this paper. The fourth part reports the temporal and spatial characteristics of China’s rural permanent population and rural electricity consumption, as well as the types of decoupling and coordination between rural permanent population and rural electricity consumption, and discusses the background reasons for the formation of the results. The fifth part is the conclusion and policy recommendations.

## 2. Literature Review

### 2.1. Research of Rural Population Outflow

Due to various reasons, the rural population continues to enter the cities for life and employment, which has a very important impact on rural development. Existing literature summarized the main reasons for rural population outflow as urbanization, high income, and the pursuit of better living conditions. In the process of urbanization, part of the rural population was directly transformed into urban population, and their living land was classified as urban land [10,11,12,13]. In addition, some areas have been urbanized by migrating rural population to urban areas, which has also reduced the number of permanent residents in rural areas [14,15,16]. On the other hand, urban areas have more employment opportunities than rural areas, and wages in urban areas are higher than in rural areas [17]. Income level not only affects quality of life and well-being, but also affects people’s social status [18]. In order to obtain high incomes, some rural people moved to cities to work and settle down, which was an important reason for the decrease in the majority of the permanent population in China’s rural areas [19,20]. In addition, urban medical care, education, transportation, and other conditions were significantly better than rural areas, so part of the rural population moved to cities to enjoy a better quality of life [20,21,22,23]. Some scholars have also found that geographical conditions have an impact on the flow of population [24]. Superior traffic conditions have reduced the cost of population mobility, thereby promoting rural population mobility [9,25].

Existing scholars have also analyzed the impact of rural population outflow. More scholars paid attention to the impact of rural population exodus on land. For example, Huang et al. (2020) took the rural areas around the Ganjiang River Basin as the research object, and pointed out that due to the outflow of rural population, a large number of rural land was idle, resulting in a waste of land resources [26]. Zhao et al. (2018) took the rural areas of Shandong as the research object. They found that due to urbanization, the rural population in Shandong Province has been greatly reduced, and the available arable land in rural areas has also decreased. However, urbanization has accelerated the innovation and dissemination of agricultural technology, which has contributed to the growth of agricultural output in Shandong [27]. Some scholars have also found that population was not only an important factor in production but also the impact of rural population loss on rural economic development. For example, Feng (2019) pointed out that due to the siphon effect of urbanization, rural labor transferred to cities, which reduced the level of resource accumulation in rural areas and led to slow rural economic development. In addition, some scholars have found that although urbanization has led to a decrease in the rural population, the technological progress effect brought about by urbanization can promote the reduction of per capita energy consumption and improve the conversion of people from traditional fuels to commercial fuels [28,29].

### 2.2. Research of Rural Electricity Consumption

Existing literature points out that the main reasons for the increase in rural electricity consumption can be summarized as improvements in power supply infrastructure, increases in rural population income, and the spread of household appliances. Good power supply infrastructure is a prerequisite for electricity. Wang et al. (2022) pointed out that the lighting area in rural China is vast, but the government and enterprises have long ignored the development of available lighting resources in rural China. In recent years, the rapid development of photovoltaic power generation technology has prompted China to accelerate the construction of photovoltaic power supply infrastructure [30]. In order to reduce poverty, the Chinese government has implemented a photovoltaic poverty alleviation policy in rural areas, which has changed the power supply conditions in rural areas, thereby promoting an increase in rural electricity consumption. Agrawal S (2020) found that rural electricity consumption in rural India increased rapidly due to the improvement of the power supply system in rural India. The improvement of income level was also an important prerequisite for the increase of electricity consumption in China’s rural areas. For a long time, due to the single source of income in China’s rural areas, the income level of farmers was relatively low and the rural electricity price was relatively high, so the rural electricity consumption was relatively low [31]. The Chinese government has increased the income of the rural population through poverty alleviation policies and agricultural subsidies, which provides an important guarantee for the rural population to use electricity [30]. In addition, in order to improve the living standards of the rural population, the Chinese government has implemented a number of policies that benefit the people, such as home appliance subsidies and home appliances to the countryside, which encouraged the rural population to buy home appliances at low cost, thus boosting the rural population’s electricity consumption [32].

### 2.3. Research on the Relationship between Rural Population and Electricity Consumption

Existing literature has also done some research on the relationship between the rural permanent residents and the rural electricity consumption. Most scholars build linear regression models to investigate the impact of rural population changes on electricity consumption from an empirical perspective. Yang et al. (2019) found that China’s rural population was rapidly moving to towns due to urbanization, which reduced the number of the rural population. However, urbanization promoted technological progress through factor agglomeration, which was conducive to promoting infrastructure improvement. Rural electricity consumption in China has long been constrained by backward power supply infrastructure. In order to improve the living conditions of rural population, the government has been promoting the construction of power supply infrastructure in new rural areas. The National Development and Reform Commission has clearly proposed water, electricity, and road co-construction projects, which has greatly improved the rural power supply and adultery, thus promoting the growth of rural power consumption [3]. Michieka et al. (2020) studied the relationship between rural and urban populations on electricity consumption in five sub-Saharan countries from 1971 to 2013 and found that the impact of rural population changes on electricity consumption in different countries is not heterogeneous [33]. There was a negative relationship between population and electricity consumption in rural areas of the Republic of Congo, but rural population changes in Kenya and South Africa have no significant impact on rural electricity consumption. Han et al. (2021) studied the social structural factors of electricity consumption in Egypt from 1997 to 2018 and also found that an increase in the rural population reduced electricity consumption [34]. Bekhet et al. (2011) analyzed the elasticities of electricity consumption for rural and urban areas in Malaysia from 1980–2009. The results showed that rural electricity consumption was inelastic due to their low income level and low appliance penetration rate. However, the regression results were not significant, indicating that electricity changes were less sensitive to rural population changes in rural areas [35]. 

We found that scholars have made some research results on the relationship between rural permanent population and electricity consumption in China, India, Africa, Southeast Asia, etc. However, most literature uses classical linear models for qualitative analysis, but lacks quantitative thinking. In addition, the existing literature examining the relationship between rural resident population and rural electricity consumption is scarce. Due to the significant differences in the level of economic development and natural conditions among provinces in China, it is still necessary to further investigate the relationship between changes in rural population in China and changes in rural electricity consumption.

## 3. Data and Methods

### 3.1. Data

China’s rural population data come from China Statistical Yearbook (2007–2020). China’s rural electricity consumption data come from China Energy Statistics Yearbook (2007–2020).

### 3.2. Methods

#### 3.2.1. Decoupling Model

Based on the decoupling theory, this study employed the decoupling index calculation model and Tapio’s [36] decoupling coefficient definition table to construct a decoupling model represented by the ratio of the average annual growth rate of rural electricity consumption to the average annual growth rate of rural permanent population. The relationship between rural electricity consumption and rural permanent population from 2007 to 2020 was analyzed. The decoupling coefficient was used to reflect whether rural electricity consumption is in line with the transition of rural permanent population. The formula is as follows:

(1) $$\alpha_{\left(n+1\right)}\;=\;\frac{(E_{\left(n+1\right)}-E_{n})/E_{n}}{(L_{\left(n+1\right)}-L_{n})/L_{n}}$$

where *n* is the nth year; $\alpha_{\left(n+1\right)}$ is the decoupling coefficient in *n* + 1 years; $E_{\left(n+1\right)}$ is the rural electricity consumption in *n* + 1 years; $E_{n}$ is the rural electricity consumption in *n* years; $L_{\left(n+1\right)}$ is the number of rural permanent residents in *n* + 1 years; $L_{n}$ is the number of rural permanent residents in year *n*.

According to Li et al. [37] and Tapio, the decoupling system values of 0.8 and 1.2 can be used as the basis for dividing the decoupling state, and the evolution characteristics of the second electricity consumption in each province can be divided into eight categories (Table 1):

(1) Strong decoupling refers to the increase of rural permanent population and the decrease of rural electricity consumption;
(2) Weak decoupling means that the number of rural permanent population and rural electricity consumption have increased, and the growth rate of rural electricity consumption is lower than that of rural permanent population;
(3) Expanding linkage refers to the similar increase in rural electricity consumption and rural population;
(4) The negative decoupling of expansion refers to the increase of rural electricity consumption and rural population, and the growth rate of rural electricity consumption was faster than that of rural permanent population;
(5) Strong negative decoupling refers to the decrease of rural permanent population and the increase of rural electricity consumption;
(6) Weak negative decoupling means that both the number of rural permanent households and rural electricity consumption were decreasing, and the reduction rate of rural electricity consumption was slower than that of rural permanent residents;
(7) Recession linkage refers to the reduction of rural electricity consumption and the rural permanent population to a similar extent;
(8) Recession decoupling means that the number of permanent residents in rural areas and rural electricity consumption decrease, and rural electricity consumption decreases faster than the population. The decoupling classification is shown in the following table:

#### 3.2.2. Coordination Degree Model

This paper adopted the coordination degree model to reflect the coordination degree within the system, which can reflect the trend from disorder to order. The coordination degree between rural electricity consumption and rural permanent population is an index to measure the coordination degree. This study introduced a coordination model to analyze the coordination relationship between rural electricity consumption and rural research population in China. The coordination model is as follows:

(2) $$C_{xy}\;=\;(x\;+y)/\sqrt{x^{2}{+\;y}^{2}}$$

where x is the annual average change rate of rural electricity consumption; y is the annual average change rate of rural permanent population; $C_{xy}$ is the coordination degree between rural electricity consumption and rural permanent population. The $C_{xy}$ value is between −1.414 and 1.414. The types of coordination degree between rural electricity consumption and rural permanent population are shown in Table 2 below. 

## 4. Results and Discussion

### 4.1. Temporal Change in Rural Permanent Population and Electricity Consumption from 2007 to 2020

**Figure 1:**
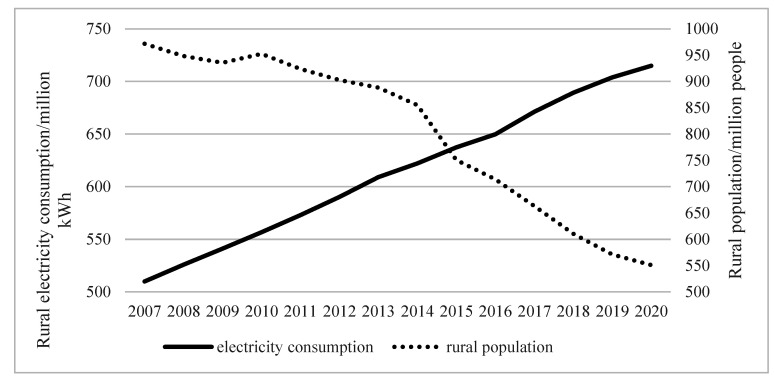
Change trend of rural permanent population and electricity consumption from 2007 to 2020.

From 2007 to 2020, China’s rural permanent population showed a downward trend (Figure 1). The rural permanent population dropped from 972 million in 2007 to 551 million in 2020, a total decrease of 421 million, an average annual decrease of 32.38 million, an average annual decrease of 4.21%. In different periods, the reduction rate of rural permanent population was different. Rural permanent population decline was slow from 2007 to 2014. The number of permanent residents in rural areas decreased by an average of 16.71 million per year, with an average annual decline rate of 1.80%. After 2014, the reduction of rural permanent population accelerated, and the rural permanent population decreased by 50.66 million, with an annual average decline rate of 6.56%. There were multiple reasons that have shaped the continued decline of China’s rural permanent population from 2007–2020. Urbanization, obtaining high income, and enjoying high-quality living conditions in cities were important reasons for the continuous decline of the rural population [38,39,40,41]. In addition, the accelerated pace of China’s agricultural reform, such as the promotion and application of mechanized production, has generated a large number of idle farmers, which has forced the rural permanent population to move into urban areas for employment [42,43]. The reason for the accelerated decline in the rural population in China in 2014 was the separation of rural land ownership, contract rights, and management rights promulgated by the central government. The “separation of powers” made the rural land enter the market economy to participate in competition, which activated the vitality of rural land and increased the direct income and leisure time of rural areas [44,45,46]. Therefore, the rural population was more willing to contract out the land, and entered the city for employment.

Electricity consumption in rural areas continued to grow from 2007 to 2020. The electricity consumption in rural areas increased from 550.99 million kWh to 971.720 million kWh, a total increase of 205.04 million kWh, an average annual increase of 15.77 million kWh, an average annual increase of 2.64%. Although China has been accelerating its urbanization process, the rural population has been pouring into the cities. However, rural electricity consumption did not decrease due to the reduction of rural population, but instead showed an upward trend. The reasons for this phenomenon were also diverse. On the one hand, the Chinese government has implemented policies that benefit farmers, such as land transfer and agricultural subsidies, which have increased the income of China’s rural permanent population, thereby enhancing the purchasing power of rural electricity [47,48]. On the other hand, China’s rural power supply infrastructure has been continuously improved, which provides the basic conditions for rural electricity consumption [49]. In addition, the Chinese government has continued to increase subsidies for the purchase of electrical appliances, which have contributed to the increasing popularity of home appliances in rural areas [50]. The above-mentioned reasons have formed an unbalanced state of China’s declining rural permanent population and rising rural electricity consumption, indicating a significant waste of power resources.

### 4.2. Spatial Changes in Rural Permanent Population and Electricity Consumption from 2007 to 2020

**Figure 2:**
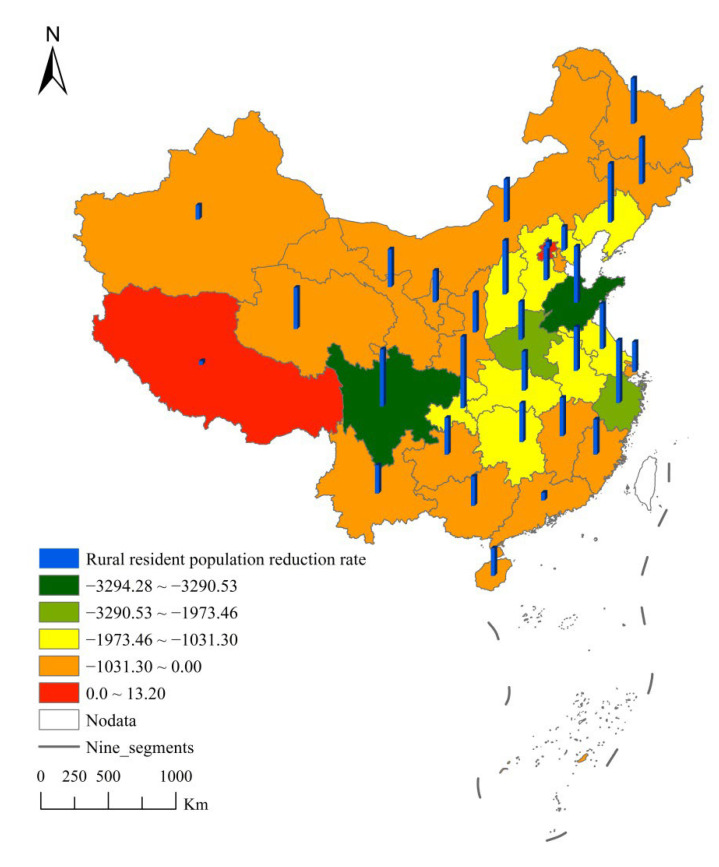
Spatial pattern of absolute increase and average annual decrease rate of rural permanent population from 2007 to 2020.

From 2007 to 2020, except Beijing and Tibet, China’s rural permanent population has been declining. This paper used the natural breakpoint method that comes with ArcGIS to divide the rural permanent population reduction of 31 provinces in China into five groups. Figure 2 shows:

Rural permanent populations in Shandong and Sichuan have fallen sharply, ranking first echelon. In the past 13 years, Shandong’s rural permanent population has decreased by 32.9428 million, and Sichuan’s rural permanent population has decreased by 32.9053 million. The cumulative reduction of the two rural permanent population account for 25.32% of the total reduction of the national rural permanent population. Urbanization and the pursuit of better living conditions were traditional reasons for rural population decline in Shandong and Sichuan provinces [51,52]. Out-of-home employment was the main reason for the decrease in the rural permanent population [53]. Both Shandong and Sichuan had a population of more than 100 million, with a high proportion of rural population and a large base of resident population in rural areas. Shandong and Sichuan were also provinces with developed agriculture. During the process of agricultural reform, farmers in these two provinces were first affected. The mechanization of agricultural production has prompted the production of a large number of idle labor in these two provinces. Shandong is located in the eastern part of China, and the rural transportation conditions are convenient, which reduces the cost of the rural population going out for employment. As a consequence, the rural population in Shandong Province has decreased the most. In recent years, the government has continuously improved the traffic conditions in rural areas, which has prompted more and more Sichuan rural people to go to the eastern coast for employment.

The rural permanent population of Henan and Zhejiang has decreased significantly, ranking second echelon. In the past 13 years, the rural permanent population in Henan has decreased by 20.51 million, and the rural permanent population in Zhejiang has decreased by 19.7346 million. The cumulative reduction of the two rural permanent residents accounted for 14.38% of the total reduction of the national rural permanent population. The reasons for the decrease in the rural permanent population in the two provinces were different. Henan was a province with a population of more than 100 million, and its rural population base was large. Since the development of the rural economy in Henan mainly relies on the agriculture, there were relatively few jobs in rural areas. In recent years, Henan has intensified its agricultural reforms, such as speeding up agricultural mechanized production, which has further produced a large number of idle farmers. In order to obtain incomes, a number of rural people in Henan had to go out to work in coastal provinces. In recent years, Henan has also accelerated the process of urbanization, which has prompted some rural population to migrate to cities. The reasons for the decline of rural population in Zhejiang were different from those in Henan. For a long time, in order to promote people’s common prosperity, Zhejiang has established small workshop-style enterprises to promote employment and increase people’s income, which has accelerated the process of urbanization. Therefore, rural permanent population of Zhejiang has been greatly reduced. 

Provinces with a moderate decrease in the rural permanent population include Liaoning, Hebei, Shanxi, Jiangsu, Anhui, Hubei, and Hunan, ranking third echelon, which decreased by 11.365 million, 11.69 million, 10.6018 million, 13.6343 million, 14.68 million, 10.313 million, and 13.1253 million, respectively. The population reduction in the five provinces accounted for 26.45% of the national rural population reduction. The reduction of rural population in these provinces ranks third echelon in the reduction of rural permanent population in China. Urbanization and the pursuit of a high quality of life in cities were part of the reason for the decline in the rural permanent population in these provinces. Out-of-home employment was an important reason for the decline of the rural permanent population in these provinces. On the one hand, there were many jobs in the urban areas of these provinces, which absorbed part of the rural population. On the other hand, these provinces are adjacent to the eastern provinces, which are the most market-oriented regions in China with a large number of jobs. In addition, the rural areas of these provinces have convenient transportation, so many rural people go out to work.

The fourth echelon is the provinces with a soft decrease in the rural permanent population, including Tianjin, Inner Mongolia, Jilin, Heilongjiang, Shanghai, Fujian, Jiangxi, Guangdong, Guangxi, Hainan, Chongqing, Guizhou, Yunnan, Shaanxi, Gansu, Qinghai, Ningxia, Xinjiang, which decreased by 520,000, 4.288 million, 5.5596 million, 6.679 million, 892.3 thousand, 5.39 million, 8.417843 million, 2.25 million, 7.44 million, 1.2039 million, 14.3495 million, 7.9932 million, 7.296 million, 7.26 million, and 5.48 million, accounting for 31.67% of the decrease in China’s rural permanent population. Inner Mongolia, Jilin, Heilongjiang, Fujian, Jiangxi, Guangxi, Hainan, Chongqing, Guizhou, Yunnan, Shaanxi, Gansu, Qinghai, Ningxia, and Xinjiang had backward rural economies, and most of the rural population was mainly engaged in agriculture. One of the important characteristics of the rural areas in these provinces was the backward traffic conditions, which not only restricted the travel of the rural population but also led to asymmetric employment information, so there were fewer people going out for employment. On the contrary, Tianjin, Shanghai, and Guangdong are located in the eastern region with a small rural permanent population base. Many enterprises in these provinces settled in the suburbs in order to reduce the cost of land use, and the rural population can be employed on the spot. Therefore, the reduction of the rural population was small. It is worth mentioning that the rural permanent population in China’s Beijing and Tibet has experienced positive growth. Beijing’s rural permanent population increased by 132,000, while Tibet’s rural permanent population increased by 72,700. Beijing had excellent conditions in many aspects, such as developed economy and small urban-rural gap. However, Beijing’s urban areas are characterized by high housing prices and congested traffic, while housing prices in rural areas are low. The distance between urban and rural areas is close and the transportation is very convenient. As a result, more and more people immigrated to live in rural areas, leading to an increase in the number of the permanent population in Beijing’s rural areas. On the contrary, the transportation in rural areas of Tibet was very backward, and the traffic cost of the rural population was very high, which restricted the flow of the rural permanent population. The natural conditions in Tibet were harsh, and most of the rural population were engaged in animal husbandry for income. There were less employment opportunities in urban areas of Tibet, which led to the rural population being more willing to stay in rural areas. In addition, the rural population had a tradition of “living together with families”, which also contributed to the continuous growth of the rural population in Tibet.

**Figure 3:**
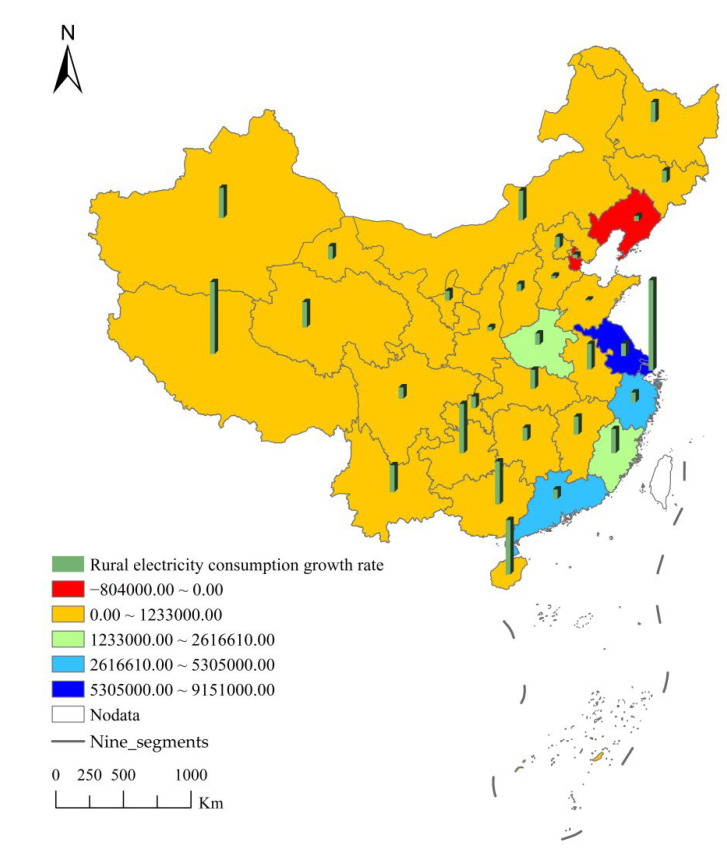
Spatial pattern of the absolute growth rate and the average annual growth rate of rural electricity consumption from 2007 to 2020.

From 2007 to 2020, except Liaoning and Tianjin, rural electricity consumption in most provinces of China continued to grow. The increase in electricity consumption varied from province to province. As shown in Figure 3, the areas with serious increase in rural electricity consumption were Jiangsu and Shanghai. From 2007 to 2020, Jiangsu’s rural electricity consumption increased by 85.2 trillion kWh, accounting for 20.24% of the national rural electricity consumption increase. Jiangsu was an important technology industry province and the largest industrial province in China. Both the high income of Jiangsu’s rural population and the increase in the penetration of household appliances were important reasons for Jiangsu’s rural electricity consumption. In addition, due to the limitation of urban areas, some enterprises gradually moved to rural areas, which not only promoted employment in rural areas but also led to a rapid increase in rural electricity consumption. From 2007 to 2020, Shanghai’s rural electricity consumption increased by 91.51 trillion kWh, accounting for 21.74% of the national rural electricity consumption increase. Both the high income of the rural population in Shanghai and the high penetration rate of household appliances have increased the consumption of electricity in the rural areas of Shanghai. In addition, Shanghai was the economic center of China and an important gathering place for foreign companies. The land price in Shanghai was very high. Therefore, most enterprises turned to Shanghai’s rural areas and parishes, which directly increased the electricity consumption in Shanghai’s rural areas.

The added value of rural electricity consumption in Zhejiang and Guangdong ranked second. Electricity consumption in rural areas in Zhejiang increased by 38.81 trillion kwh, accounting for 9.221% of the added value of rural electricity consumption in the country. Zhejiang was a model province of common prosperity in China, where the income of the rural population continued to increase, resulting in the increasing purchasing power of electricity for the rural population. The penetration rate of household appliances in rural areas in Zhejiang was high, so rural electricity consumption had increased. Electricity consumption in rural areas of Guangdong increased by 53.05 billion kwh, accounting for 12.60% of the added value of rural electricity consumption in the country. Since Guangdong was a major manufacturing province of household appliances in China, the penetration rate in rural areas of Guangdong had grown rapidly, which directly increases the electricity consumption in rural areas. In addition, in order to achieve economic growth, Zhejiang and Guangdong have implemented a number of policies to encourage innovation of small companies and mass company. Therefore, there were a large number of small manufacturing enterprises in Zhejiang and Guangdong. Due to the low price and high labor cost in urban areas, small and medium-sized enterprises were mostly distributed in rural areas, which greatly increased the electricity consumption in rural areas.

Henan’s rural electricity consumption ranked third in the added value of rural electricity consumption. From 2007 to 2020, Henan’s electricity consumption increased by 149.77 million kWh, accounting for 3.56% of the added value of rural electricity consumption in the country. Henan had relatively developed agriculture and was an important grain producing area in China. With the development of China’s agricultural science and technology in recent years, Henan’s agricultural production had gradually been modernized, and agricultural electricity consumption had increased rapidly.

The added value of rural electricity consumption in 22 provinces, including Heilongjiang, Jilin, Shandong, Hebei, and Anhui, ranked fourth echelon in China. A total of 161,495,770,000 kwh increased, accounting for 38.37% of the national rural electricity consumption. Most of these provinces are located in the central and western regions with large rural populations. These provinces were also large agricultural provinces, but the growth in electricity consumption was the smallest. Most of these provinces are located in the central and western regions, where the income level of the rural population grows slowly and the penetration rate of household appliances is low. Therefore, the rural electricity consumption increased slowly.

It is worth noting that the rural electricity consumption in Liaoning and Tianjin had been decreasing from 2007 to 2020. The rural electricity consumption in Liaoning decreased by 8.04 billion kWh, and the rural electricity consumption in Tianjin had decreased by 1152.2 million kWh. With the acceleration of urbanization, Tianjin’s rural areas had been gradually incorporated into urban construction, and some rural areas had become urban areas, which reduces rural electricity consumption. Liaoning was an important province for brain drain in China. In particular, the rural population had entered the city for employment and settlement, which greatly reduced the electricity consumption in the countryside. In addition, the “new rural construction” strategy in Tianjin and Liaoning concentrates on the rural population and optimizes the rural power grid installation, which also reduces rural power consumption.

### 4.3. The Relationship between Rural Electricity Consumption and Rural Permanent Population from 2007 to 2020

In order to understand the relationship between rural permanent population and electricity consumption in China, this paper established a decoupling model and a coordination model between rural electricity consumption and rural permanent population. By reporting and discussing the results of the two models, the decoupling relationship between them can be fully reflected. According to the decoupling model and coordination model, the decoupling coefficient and coordination degree between rural electricity consumption and rural permanent population from 2007 to 2020 were calculated, as shown in the following table.

According to the decoupling theoretical model, from 2007 to 2020, the changes in the decoupling relationship between rural electricity consumption and rural permanent population showed three states: expanding negative decoupling, recession link, and strong negative decoupling. From a national perspective, the decoupling coefficient between rural permanent population and rural electricity consumption in 2007–2020 was −0.6168 (Table 3), which was a strong negative decoupling, indicating that the total rural population was declining and electricity consumption was increasing. The reduction rate of rural permanent population was greater than the growth rate of rural electricity consumption. At the provincial level, Beijing and Tibet belonged to the expanding negative decoupling. The rural permanent population and rural electricity consumption were both increasing, and the growth rate of rural electricity consumption was greater than that of the rural permanent population. However, the reasons for the expanding negative decoupling in the two places were indeed different. Beijing’s economy was very developed, housing prices were high, and the urban area was densely populated, resulting in the phenomenon of “anti-urbanization”. On the contrary, Tibet was economically backward, with poor natural conditions and inconvenient transportation, which reduces the mobility of the rural population. Tianjin and Liaoning belonged to the recession link. Both rural permanent population and rural electricity consumption were decreasing, but the reduction rate of the former was slower than that of the latter. The other 27 provinces, including Hebei, Shanxi, and Shandong, were strong negative decoupling areas. In conclusion, the rural electricity consumption coordination areas and the rural permanent population coordination areas in 27 provinces showed strong negative decoupling to varying degrees. In fact, with the rapid reduction of the rural permanent population, rural electricity consumption was increasing, resulting in a serious waste of power resources.

According to the coordination degree model, the coordination degree between national rural permanent population and rural electricity consumption was −0.3262. National rural permanent population showed negative growth, and the rural electricity consumption showed positive growth, which was uncoordinated. Focusing on the provincial level, there were three types across the country: reconcilable, basically coordinated, and uncoordinated.

The reconcilable provinces included Shanghai, Inner Mongolia, Jilin, Jiangsu, Anhui, Fujian, Jiangxi, Heilongjiang, Henan, Hubei, Hunan, Guangdong, Guangxi, Hainan, Guizhou, Yunnan, Gansu, Qinghai, Ningxia, and Xinjiang. The resident rural population in these provinces has decreased, while rural electricity consumption has increased. The rate of decrease in the rural resident population was higher than the growth rate of rural electricity consumption, but the reasons varied from province to province. Urbanization in Shanghai and Jiangsu has played a large role in reducing the rural population. Both Jiangsu and Shanghai have been expanding their urban areas by incorporating rural areas and converting rural populations into urban populations, which has significantly reduced the resident rural population. A remarkable fact was that Shanghai and Jiangsu are located in the Yangtze River Delta metropolitan area with more employment opportunities in urban areas, and the rural population was willing to move to the city for stable jobs to increase income. In addition, the transportation in Shanghai and Jiangsu was very developed and the level of marketization was relatively high, which reduced the transportation cost and information search cost of rural population transferring to urban areas. Therefore, rural permanent population was more willing to transfer to urban areas. However, despite rural electricity consumption in these two regions having increased significantly due to the popularity of household appliances and the rise in income levels of the rural population, the growth rate of electricity consumption was smaller than the reduction rate of rural population, which leads to the current situation of reconciliation between rural population and electricity consumption. For the other provinces, although urbanization played a weak role in reducing the rural resident population, out-migration for employment significantly reduced the number of rural residents in these provinces. However, the rugged terrain in rural areas of these provinces was not conducive to building electricity supply infrastructure, which limited access to electricity. In addition, the slow growth of farmers’ income levels in these provinces had resulted in low purchasing power in rural areas of these provinces and slow growth of electricity consumption in these areas. As a result, the resident population in rural areas was decreasing faster than the increase in electricity consumption.

The basic coordination areas included Beijing and Tibet. The growth rate of Beijing’s rural permanent population from 2007 to 2020 was lower than that of rural electricity consumption. The average annual growth rate of Beijing’s rural permanent population was 0.38%, and the average annual growth rate of rural electricity consumption was 4.1%. The coordination degree was 1.0885. The growth rate of rural permanent residents in Tibet from 2007 to 2020 was lower than that of rural electricity consumption. The growth rate of the rural permanent population in the Tibet was 0.24%, the average annual growth rate of rural electricity consumption was 13.56%, and the coordination degree was 1.0178. Although the coordination degree between Beijing and Tibet was close, they represented completely different types of regions. There were obviously differences in many aspects such as economic development level, production level, and lifestyle. The growth rate of rural permanent population in Beijing was 1.58 times that of Tibet. However, the growth rate of rural permanent population in Beijing was much higher than that in Tibet. The growth rate of electricity consumption in rural Tibet was 56.5 times that of Beijing, but the growth rate of electricity consumption in Beijing was higher than that in Tibet. Beijing has a developed economy with an urbanization level of 86.60%, ranking first in China. In recent years, due to the gradual shrinking of the usable range in cities and towns, scientific research institutions and productive enterprises have moved to suburban areas and rural areas. This has not only led to the transfer of some population to rural areas, resulting in an increase in the number of rural populations, but also increased rural electricity consumption. On the contrary, Tibet was economically backward and has a low level of urbanization. Animal husbandry was an important source of income in Tibet. The rural areas of Tibet had vast usable land and grasslands. Therefore, the majority of the population remained in rural areas. In addition, Tibet’s high altitude and poor natural conditions in most areas further reduced the flow of people. In recent years, the government had not only stepped up poverty alleviation efforts in rural areas of Tibet to increase their income, but also continuously improved the construction of electricity infrastructure in rural areas of Tibet. The number of household appliances in rural areas of Tibet has increased rapidly. As a result, electricity consumption in rural Tibet has increased rapidly.

The uncoordinated regions included Tianjin, Hebei, Shanxi, Liaoning, Zhejiang, Shandong, Sichuan, Chongqing, and Shaanxi. The rural permanent population in these provinces decreased, while rural electricity consumption increased. However, the reduction rate of the rural permanent population was less than the growth rate of rural electricity consumption. The reasons for the change varied from province to province. Urbanization is an important reason for the decrease in the rural resident population in Tianjin and Liaoning. In addition, due to the convenient transportation in Tianjin and Liaoning, the rural population has been employed outside the countryside, which has further reduced the rural resident population. At the same time, Tianjin and Liaoning accelerated the implementation of the “new rural construction” strategy and concentrated on the resettlement of the rural population, which led to more efficient use of electricity and thus reduced rural electricity consumption. The rate of reduction in rural resident population is not consistent with the rate of reduction in rural electricity consumption, resulting in a mismatch between rural population and electricity consumption in the two provinces. Coal-fired and hydroelectric power generation are the main methods of electricity generation in China. Abundant coal resources in Shanxi, Shaanxi, Shandong, and Hebei, and abundant hydro resources in Zhejiang, Sichuan, and Chongqing provide the prerequisites for rural electricity supply. In addition, Zhejiang, Shandong, and Hebei have developed rural economies with increasing penetration of household appliances. As the income level of the rural population increases, rural electricity consumption is rapidly increasing. Shanxi, Shaanxi, Sichuan, and Chongqing have a large rural poverty population and are key regions for the government to implement poverty alleviation measures. The government set up special poverty alleviation funds for these regions to increase the income of the poor in the form of direct subsidies, which expanded the purchasing power of the rural population for electricity and led to a rapid increase in rural electricity consumption. As a result, the reduction rate of rural population is smaller than the growth rate of rural electricity, which appears incongruous. 

Combining the decoupling model and the coordination degree model can more accurately analyze the coordination degree of rural population and electricity consumption. This paper used the natural discontinuity method to divide the strong negative decoupling and non-coordinated provinces into high quality strong non-coordinated provinces and moderate to mildly strong non-decoupling non-coordinated provinces and explored the degree of strong negative decoupling in these 10 provinces. The results show:

(1) Strong negative decoupling provinces, including Shanxi, Zhejiang, Chongqing, and Sichuan provinces and regions, showed strong negative decoupling. The average reduction rate of permanent rural populations in these provinces was 5.39%, much higher than the national average reduction rate (4.27%). The average annual electricity consumption growth rate in these provinces was 3.68%, up from the whole country’s 2.68%. The large loss of rural permanent population and the growth of rural electricity consumption were the main reasons for the disharmony between rural population and electricity consumption in these provinces.

(2) Hebei, Shandong, and Shaanxi belonged to the medium strong negative decoupling groups. The average reduction rate of rural permanent population in these three provinces is 3.70%, lower than the national average of 4.27%. The average annual electricity consumption growth rate in these provinces was 1.09%, down from the national 2.68%. The deviation between the growth rate of rural electricity consumption and the growth rate of national average electricity consumption was greater than that of rural population growth rate, which was the most important reason for the high negative decoupling between rural population and electricity consumption in these provinces.

There were multiple reasons that shaped the change in China’s rural resident population and rural electricity consumption from 2007 to 2020. The combination of diversified causes shaped an imbalance between the decrease in rural resident population and the increase in rural electricity consumption in China. From a provincial perspective, diversified causes also played different roles in reducing the rural permanent population and increasing rural electricity consumption, which created heterogeneity across provinces in terms of decreasing rural permanent population and increasing rural electricity consumption, leading to different types of decoupling and coordination across provinces. 

By further analyzing and summarizing the causes of urbanization and urban employment in each province, this paper found that the attributes of these causes were influenced by the macro conditions of the rural areas themselves and thus affected the changes in the rural population. For example, the population base and geographical conditions determine the different roles played by these causes. In terms of the rural population base, the provinces with more rural populations were those with more decreasing rural resident population, such as Sichuan, Henan, Shandong, and Zhejiang. Agricultural reform affected the large rural population provinces first by reducing the use of farmers. The rural population in these areas moved to the cities for employment in order to obtain higher income. Provinces with easy access to rural transportation were those with a higher reduction of rural population, such as Shandong, Hebei, Liaoning, Jiangsu, and Zhejiang, the central and eastern provinces of China. Developed transportation conditions not only reduced the asymmetry of employment information, but also reduced the transportation costs of the rural population. Similarly, the reasons affecting the changes in rural electricity consumption were influenced by rural macro conditions. From the perspective of power generation resource endowment, abundant coal and hydro resources provided the basic conditions for rural electricity supply in these provinces. Electricity consumption in rural areas was higher in provinces rich in coal and hydro resources, such as Shaanxi, Shanxi, Shandong, Hebei, and Zhejiang. Areas with high income levels of rural population had greater growth in electricity consumption, for example, Jiangsu, Zhejiang, Shanghai, and Guangdong. The growth of electricity consumption is higher in rural areas with high penetration of home appliances, for example, Jiangsu, Shanghai, Zhejiang, and Guangdong. The macro conditions in rural areas determine the causes of changes in rural resident population and changes in electricity consumption, thus affecting the decoupling and harmonization relationship between rural population and electricity consumption. It is worth noting that the role of China’s “agriculture, rural areas, and farmers” policy (Three rural policies) in rural China cannot be ignored. In order to improve the income levels and life satisfaction of the rural population, the Chinese government is constantly improving its “Three rural policies”. It is clear that China’s “Three rural policies” have had a large impact on rural population and electricity consumption. Agricultural production reforms have generated a large number of idle farmers, agricultural subsidies have increased farmers’ income, and subsidies for home appliance purchases have increased the rural population’s demand for electricity consumption. As a result, China’s “Three rural policies” affected the coupled relationship between rural resident population and electricity consumption by influencing changes in the population and changes in electricity consumption.

## 5. Conclusions and Suggestions

Based on the comprehensive perspective of the relationship between rural permanent population and rural electricity consumption, this study used decoupling model and coordination model to analyze the temporal change characteristics, spatial variation characteristics, and coupling coordination relationship between the national and provincial rural permanent population and electricity consumption. The main conclusions are as follows: (1) Time-Wise, from 2007 to 2020, China’s rural permanent population continued to decrease, while the rural electricity consumption continued to increase. Spatially, the rural permanent population continued to decrease in all provinces except Beijing and Tibet. Rural electricity consumption continued to increase in all provinces except Tianjin and Liaoning. (2) In terms of the type of decoupling, there was a negative decoupling between China’s rural permanent population and rural electricity consumption. At the provincial level, Beijing and Tibet belonged to the expanding negative decoupling, Tianjin and Liaoning belonged to the recession link, and the other 27 provinces, such as Hebei, Shanxi, Shandong, etc., were strong negative decoupling. (3) In terms of the coordination type, China’s rural permanent population and rural electricity consumption was uncoordinated. At the provincial level, the regions that can be coordinated include 20 provinces, including Shanghai, Inner Mongolia, Jilin, Jiangsu, Anhui, Fujian, and Jiangxi, etc. The basic coordination areas included Beijing and Tibet. The uncoordinated regions included Tianjin, Hebei, Shanxi, Liaoning, Zhejiang, Shandong, Sichuan, Chongqing, and Shaanxi. (4) In terms of the comprehensive degree of decoupling and coordination, Shanxi, Zhejiang, Chongqing, and Sichuan showed extremely strong negative decoupling, while Hebei, Shandong, and Shaanxi belonged to moderately strong negative decoupling groups.

China’s rural population continued to flood into cities for settlement and employment, which contributed to social and economic development. However, rural electricity consumption had not decreased with the reduction of rural permanent population. There was a certain harm in the phenomenon of “people decrease and electricity increase” in China’s rural areas. First, most of the electricity supply was coal-fired, which further exacerbated the consumption of conventional natural energy and may pollute the environment. Second, the increase in rural electricity consumption had also intensified government investment in infrastructure in rural areas, which may crowd out other uses of resources, increase resource misallocation, and lead to unfair resource allocation. Various regions in China are faced with the task of energy conservation and emission reduction, which raises the requirements for electricity usage in various regions. Some recommendations are suggested as follows: (1) The government should strengthen the supervision of electricity consumption in rural areas and establish the ladder price of rural electricity to prevent waste of power resources. (2) Accelerate the construction of rural power infrastructure, especially photovoltaic power generation infrastructure, to provide clean power supply sources for rural areas. (3) Considering that the Chinese government has continuously implemented the policy of sending home appliances to the countryside, the government should encourage enterprises to develop household appliances with low power consumption and high performance, and increase their promotion in rural areas to save the use of power resources. (4) Considering that the rural population has little awareness of saving electricity, the government should strengthen the publicity and education of saving electricity, thus cultivating the habit of saving electricity.

### Limitations

Although this paper analyzed the decoupling and coordination of rural permanent population and rural electricity consumption in each province of China, there are still some shortcomings. On the one hand, the research method of this paper is relatively simple. There are many research methods on the coupling coordination relationship in the existing literature. The changes between rural population and rural electricity consumption can be further verified by selecting other suitable coupling coordination methods. On the other hand, the rural electricity consumption in this paper is the overall rural electricity consumption. However, electricity use in rural areas can be divided into industrial and residential electricity. Due to the limitation of data, this paper cannot distinguish rural residential electricity consumption from industrial electricity consumption. The above is also the direction for further improvement by the author in the future.

## Figures and Tables

**Table 1:** Definition of degree of decoupling.

Decoupling State		RE	RL	α
Negative decoupling	Expansion and negative decoupling	>0	>0	>1.2
	Strong negative decoupling	>0	<0	<0
	Weak negative decoupling	<0	<0	0 < α < 0.8
Decoupling	Weak decoupling	>0	<0	0 < α < 0.8
	Strong decoupling	<0	>0	<0
	Recession decoupling	<0	<0	>1.2
Link	Expansion contact	>0	>0	0.8 < α < 1.2
	Recession contact	<0	<0	0.8 < α < 1.2

**Table 2:** The Type of Coordination Degree.

C_xy_	x, y	Coordination Degree Type
C_xy_ = 1.414	x = y, and x > 0, y > 0	More coordinated
1.2 ≤ C_xy_ < 1.414	x ≈ y	Coordinated
1.0 ≤ C_xy_ < 1.2	x > 0, y > 0 and x > y	Basically coordinated
0.5 ≤ C_xy_ < 1.0	x > 0, y < 0	Reconcilable
−1.414 ≤ C_xy_ < 0	x < 0, y < 0, or x > 0, and y < 0	Uncoordinated

**Table 3:** Decoupling coefficient and coordination between rural electricity consumption and rural residents.

Province	Rural Permanent Population Growth Rate (%)	Growth Rate of Electricity Consumption (%)	Decoupling Coefficient	Type of Decoupling	Coordination Degree C_xy_	Type of Coordination	Comprehensive Type
Nationwide	−0.0427	0.0263	−0.6168	Strong negative decoupling	−0.3262	Uncoordinated	Strong negative decoupling and Uncoordinated
Beijing	0.0038	0.0410	10.7236	Expanding negative decoupling	1.0885	Basically coordinated	Expanding negative decoupling and Basically coordinated
Tianjin	−0.0167	−0.0189	1.1278	Recession link	−1.4117	Uncoordinated	Recession link and Uncoordinated
Hebei	−0.0251	0.0132	−0.5242	Strong negative decoupling	−0.4214	Uncoordinated	Strong negative decoupling and Uncoordinated
Shanxi	−0.0446	0.0283	−0.6348	Strong negative decoupling	−0.3083	Uncoordinated	Strong negative decoupling and Uncoordinated
Inner Mongolia	−0.0331	0.0807	−2.4389	Strong negative decoupling	0.5459	Reconcilable	Strong decoupling and Reconcilable
Liaoning	−0.0504	−0.0274	0.5447	Recession link	−1.3565	Uncoordinated	Recession link and Uncoordinated
Jilin	−0.0364	0.0424	−1.1655	Strong negative decoupling	0.1077	Reconcilable	Strong decoupling and Reconcilable
Heilongjiang	−0.0360	0.0621	−1.7258	Strong negative decoupling	0.3639	Reconcilable	Strong decoupling and Reconcilable
Shanghai	−0.0220	0.1524	−6.9245	Strong negative decoupling	0.8468	Reconcilable	Strong decoupling and Reconcilable
Jiangsu	−0.0358	0.0433	−1.2105	Strong negative decoupling	0.1341	Reconcilable	Strong decoupling and Reconcilable
Zhejiang	−0.0554	0.0367	−0.6618	Strong negative decoupling	−0.2821	Uncoordinated	Strong negative decoupling and and Uncoordinated
Anhui	−0.0344	0.0731	−2.1235	Strong negative decoupling	0.4787	Reconcilable	Strong decoupling and Reconcilable
Fujian	−0.0264	0.0706	−2.6767	Strong negative decoupling	0.5868	Reconcilable	Strong decoupling and Reconcilable
Jiangxi	−0.0292	0.0574	−1.9640	Strong negative decoupling	0.4374	Reconcilable	Strong decoupling and Reconcilable
Shandong	−0.0473	0.0068	−0.1438	Strong negative decoupling	−0.8475	Uncoordinated	Strong negative decoupling and Uncoordinated
Henan	−0.0288	0.0403	−1.3953	Strong negative decoupling	0.2303	Reconcilable	Strong decoupling and Reconcilable
Hubei	−0.0298	0.0597	−2.0038	Strong negative decoupling	0.4482	Reconcilable	Strong decoupling and Reconcilable
Hunan	−0.0297	0.0446	−1.5042	Strong negative decoupling	0.2791	Reconcilable	Strong decoupling and Reconcilable
Guangdong	−0.0051	0.0351	−6.8529	Strong negative decoupling	0.8451	Reconcilable	Strong decoupling and Reconcilable
Guangxi	−0.0214	0.1019	−4.7727	Strong negative decoupling	0.7737	Reconcilable	Strong decoupling and Reconcilable
Hainan	−0.0200	0.1167	−5.8379	Strong negative decoupling	0.8168	Reconcilable	Strong decoupling and Reconcilable
Chongqing	−0.0671	0.0418	−0.6232	Strong negative decoupling	−0.3198	Uncoordinated	Strong negative decoupling and Uncoordinated
Sichuan	−0.0485	0.0404	−0.8334	Strong negative decoupling	−0.1280	Uncoordinated	Strong negative decoupling and Uncoordinated
Guizhou	−0.0278	0.1104	−3.9753	Strong negative decoupling	0.7258	Reconcilable	Strong decoupling and Reconcilable
Yunnan	−0.0205	0.0757	−3.6903	Strong negative decoupling	0.7036	Reconcilable	Strong decoupling and Reconcilable
Tibet	0.0024	0.1356	55.7806	Expanding negative decoupling	1.0178	Basically coordinated	Expanding negative decoupling Basically coordinated
Shaanxi	−0.0303	0.0169	−0.5571	Strong negative decoupling	−0.3869	Uncoordinated	Strong negative decoupling and Uncoordinated
Gansu	−0.0286	0.0453	−1.5817	Strong negative decoupling	0.3109	Reconcilable	Strong decoupling and Reconcilable
Qinghai	−0.0319	0.0730	−2.2926	Strong negative decoupling	0.5168	Reconcilable	Strong decoupling and Reconcilable
Ningxia	−0.0231	0.0359	−1.5502	Strong negative decoupling	0.2982	Reconcilable	Strong decoupling and Reconcilable
Xinjiang	−0.0096	0.0816	−8.4583	Strong negative decoupling	0.8757	Reconcilable	Strong decoupling and Reconcilable
